# Untargeted Metabolomics Reveals Metabolic Stress Alleviation by Prepartum Exercise in Transition Dairy Cows

**DOI:** 10.3390/metabo12040309

**Published:** 2022-03-31

**Authors:** Zhengzhong Luo, Yixin Huang, Li Ma, Jing Jiang, Qiao Luo, Zhuo Yang, Kang Yong, Liuhong Shen, Shumin Yu, Xueping Yao, Jinzhong Tao, Suizhong Cao

**Affiliations:** 1 Department of Clinical Veterinary Medicine, College of Veterinary Medicine, Sichuan Agricultural University, Chengdu 611130, China; zhengzhongluo@163.com (Z.L.); hyxemma213@gmail.com (Y.H.); mali2021202103@126.com (L.M.); jiangjing_94@163.com (J.J.); luoqiao2016@163.com (Q.L.); shenlh@sicau.edu.cn (L.S.); yayushumin@sicau.edu.cn (S.Y.); yaoxueping74@126.com (X.Y.); 2 Institute of Biodiversity, Animal Health & Comparative Medicine, College of Medical, Veterinary & Life Sciences, University of Glasgow, Glasgow G61 1QH, UK; 3 Agriculture College, Ningxia University, Yinchuan 750021, China; Jennyyangzhuo@126.com; 4 Department of Animal Husbandry & Veterinary Medicine, College of Animal Science and Technology, Chongqing Three Gorges Vocational College, Chongqing 404100, China; yongkangkang@126.com

**Keywords:** prepartum exercise, transition dairy cow, metabolomics, metabolic stress

## Abstract

Prepartum exercise (PA) has been proposed as a strategy for the peripartum management of dairy cows; however, the mechanism by which PA affects metabolism has not been elucidated. Here, we investigated the metabolic changes in transition dairy cows with PA. Holstein transition multiparous dairy cows were assigned to an exercise (*n* = 12) or a control (*n* = 12) group; the cows in the exercise group walked for a targeted 45 min at 3.25 km/h, two times a day. Plasma non-esterified fatty acid (NEFA), β-hydroxybutyric acid (BHBA), glucose, and triglyceride levels were measured, and metabolic profiles were analyzed using untargeted mass spectrometry. Compared with those in the control group, the concentrations of NEFA at −7 d, glucose at 0 d, and BHBA at +7 d relative to calving were considerably decreased in the exercise group. Untargeted metabolomics analysis revealed differences in the levels of key metabolites, including kynurenine, tryptophan, homovanillic acid, dopamine, cis-9-palmitoleic acid, and palmitic acid, between the exercise and control group cows. This study suggests that PA may decrease homovanillic acid and cis-9-palmitoleic acid levels and increase tryptophan levels to alleviate the metabolic stress in dairy cows during calving, thereby improving postpartum health.

## 1. Introduction

The transition period, an important phase for dairy cows [1], is the 3 weeks between pre- and post-calving, representing the transition from late gestation (through calving) to lactation. Moreover, the feeding behaviors of dairy cows change during this period [2]. Specifically, the dry matter intake (DMI) decreases by 20–40% in the 3 weeks before calving and water intake rapidly increases after calving [3]. The synthesis of milk proteins, fats, and lactose increases rapidly during lactation, thereby increasing energy requirements [4]. All transition dairy cows experience different degrees of negative energy balance (NEB) which is the result of the DMI not meeting the high energy requirements [5]. Glucose, lipid, and amino acid metabolic processes are also considerably affected after calving because of physiological and nutritional changes [6]. In particular, lipolysis in the adipose tissue is intensified to adapt to the NEB, which increases the level of non-esterified fatty acids (NEFA) in the blood during the transition period [7]. This elevated production of NEFA during early lactation generates a large amount of β-hydroxybutyric acid (BHBA), which may also intensify or aggravate metabolic disorders [4,8]. Thus, strategies to alleviate the development of metabolic disorders in dairy cows during the transition period remain an important research topic.

Exercise is one of the proposed methods for optimizing peripartum management practices [9,10]. It has been reported that 1.5 h of daily exercise (3.25 km/h) during gestation could improve the physical fitness of dairy cows and maintain physiological homeostasis [11,12]. Furthermore, prepartum exercise has been found to increase the lying time of pregnant cows and reduce the lying frequency [13]. The increase in lying time could help decrease the concentration of NEFA by reducing the body condition score loss after calving, thus relieving metabolic stress [14]. Similarly, prepartum exercise twice a day (3.4 km/h) has been found to reduce lipolysis and lipid utilization [15]. Moreover, exercise during gestation improved maternal glucose metabolism and the overall metabolic health of offspring [16]. Therefore, prepartum exercise appears to have a positive effect on cows.

Researchers have applied systems biology methods to study the periparturient physiology and diseases of dairy cows [17]. Metabolomics is a branch of systems biology in which small-molecule metabolites (molecular mass < 1500 Da) can be detected in blood and urine samples to help reveal any potential physiological changes in animals under certain conditions and to identify biomarkers for the diagnosis and pathogenesis of diseases [18,19]. We hypothesized that the prepartum exercise of dairy cows during calving regulates certain metabolic pathways to alleviate metabolic stress. To test this hypothesis, an untargeted metabolomics approach was utilized to characterize the plasma metabolites in cows at −7 d, 0 d, +7 d, and +30 d relative to parturition and to identify differential metabolites and key metabolic pathways in the exercise and non-exercise (control) group cows. Moreover, the concentrations of NEFA, BHBA, glucose, and triglycerides (TG) in the plasma were compared between the exercise and control groups.

## 2. Results

### 2.1. Plasma Analyses

**Figure 1:**
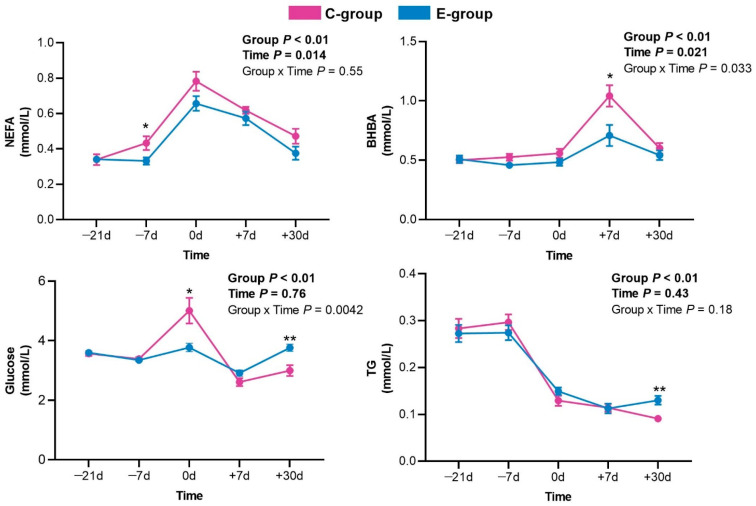
Concentrations of non-esterified fatty acids (NEFA), β-hydroxybutyric acid (BHBA), glucose, and triglyceride (TG) in the exercise (E-group) and control (C-group) groups at −21 d, −7 d, 0 d, +7 d, and +30 d relative to calving. Data are expressed as a mean ± SEM; * *p* < 0.05, ** *p* < 0.01, comparing the two groups at a given time point.

Plasma NEFA and glucose concentrations increased from −21 d to calving and subsequently decreased in the control cows (Figure 1). The plasma BHBA concentration increased from −21 d to +7 d and then declined thereafter in the control cows. Compared with that measured before calving, the TG concentration apparently decreased in the control cows after calving. In the exercise group (walked for a targeted 45 min at 3.25 km/h, twice a day), the concentration of NEFA in the plasma at −7 d, glucose concentration at 0 d, and BHBA concentration at +7 d decreased compared with those in the control cows (Figure 1). During lactation, the glucose and TG concentrations were considerably higher in the exercise group than in the control group at +30 d relative to calving.

### 2.2. Metabolic Profiles

**Figure 2:**
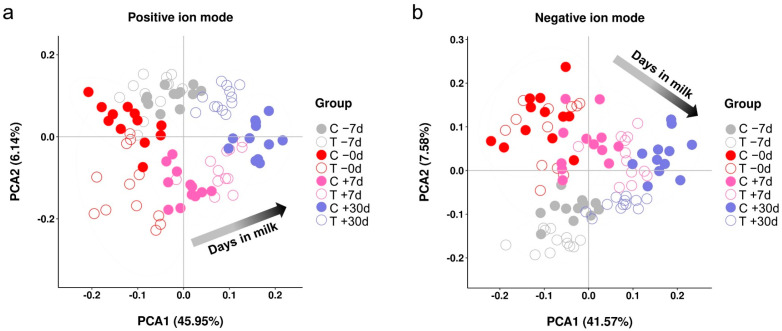
Principal component analysis score plot of the plasma metabolome for the exercise and control groups at −7 d, 0 d, +7 d, and +30 d relative to calving in the positive-ion mode (**a**) or negative-ion mode (**b**). PC1 represents the first principal component and PC2 represents the second principal component. The black arrow indicates the days in milk of dairy cows during lactation, in which, the light and dark shading represent shorter and longer days in milk, respectively.

Ultrahigh-performance liquid chromatography time-of-flight mass spectrometry (UHPLC-TOF/MS) revealed 3071 ion peaks in the positive-ion mode and 2123 ion peaks in the negative-ion mode. After pre-processing the ion peak data, multidimensional statistical analyses were performed. The principal component analysis (PCA) score plot of the exercise group versus the control group for −7 d, 0 d, +7 d, and +30 d showed that the samples were well-separated between the exercise and control groups at different time points (Figure 2). With an increase in the days in milk, the global metabolites are clearly separated. To analyze the differences among the time points in the control group, longitudinal orthogonal partial least squares-discriminant analysis (OPLS-DA) was performed. The permutation test of the model parameters in the positive- and negative-ion modes were R^2^Y = 0.924, Q^2^ = 0.891 and R^2^Y = 0.943, Q^2^ = 0.883, respectively (Supplementary Figure S1). To analyze the differences between the exercise and control groups at each time point, cross-sectional OPLS-DA was performed. The model parameters in the positive- and negative-ion modes (R^2^Y and Q^2^) are shown in Supplementary Table S1; they were > 0.4 between each group, indicating that the model was stable and reliable.

### 2.3. Differential Metabolites and Pathway Analysis

**Figure 3:**
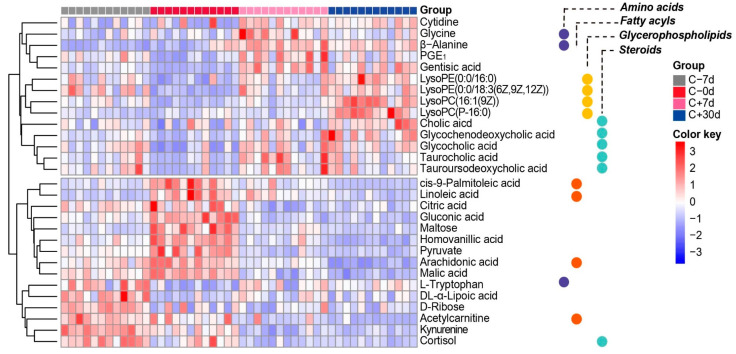
Heatmap demonstrating the dynamic changes in the plasma levels of 29 differential metabolites identified in control group cows at −7 d, 0 d, +7 d, and +30 d relative to calving. The heatmap color key represents the mean log (relative abundance of metabolite) and normalized efficacy values.

**Figure 4:**
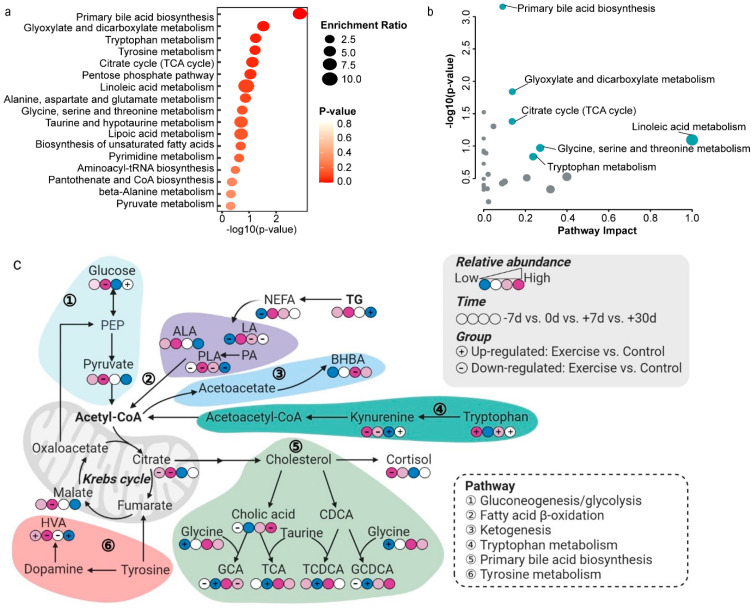
KEGG pathway analysis and dynamic changes in the plasma levels of differential metabolites. (**a**) KEGG enrichment analysis of 29 differential metabolites identified in control group cows; the circle size represents the enrichment ratio. The *p* value is represented in terms of color in which a deeper color (red) of circles represents a decreasing trend. (**b**) Pathway analysis of 29 differential metabolites identified in control group cows using topology analysis; the circle size represents the pathway impact. (**c**) Metabolic pathways involving major metabolites that differed in plasma concentration between the exercise and control cows at −7 d, 0 d, +7 d, and +30 d relative to calving. Four circles indicate the metabolites identified in control group cows at −7 d, 0 d, +7 d, and +30 d relative to calving. The color of circles indicates the relative abundance of the metabolite in which blue (red) circles represent low (high) relative abundance. The symbol “+“ indicates that the metabolite concentrations are higher in the exercise group than in the control group, and the symbol “-” indicates that the metabolite concentrations are lower in the exercise group than in the control group. NEFA = non-esterified fatty acids; BHBA = β-hydroxybutyric acid; TG = triglyceride; PEP = phosphoenolpyruvate; ALA = arachidonic acid; PLA = palmitoleic acid; LA = linoleic acid; PA = palmitic acid; HVA = homovanillic acid; GCA = glycocholic acid; TCA = taurocholic acid; CDCA = chenodeoxycholic acid; TCDCA = tauroursodeoxycholic acid; GCDCA = glycochenodeoxycholic acid. The figure was created with BioRender (https://biorender.com/).

Twenty-nine differential metabolites were identified among the control cows during the transition from −7 d, 0 d, +7 d, to +30 d relative to calving (Supplementary Table S2). These metabolites were mainly fatty acids, steroids, glycerophospholipids, and amino acids (Figure 3). The Kyoto Encyclopedia of Genes and Genomes (KEGG) enrichment and pathway impact analyses indicated that these differential metabolites mainly participate in primary bile acid biosynthesis, glyoxylate and dicarboxylate metabolism, the citrate cycle, linoleic acid metabolism, and tryptophan metabolism (Figure 4a,b).

Differential metabolites between cows in the exercise group and those in the control group were also identified, with a total of 35, 31, 24, and 33 differential metabolites identified between the groups at −7 d, 0 d, +7 d, and +30 d, respectively (Supplementary Table S3). To further analyze the dynamic changes in differential metabolites, a metabolic pathway diagram was drawn based on the KEGG pathway analysis, demonstrating that the metabolites are involved in tryptophan metabolism, fatty acid metabolism, primary bile acid metabolism, tyrosine metabolism, and glucose metabolism, which are connected through some key metabolites such as acetyl-CoA and cholesterol (Figure 4c). Compared with those during late pregnancy, primary bile acid metabolism and tryptophan metabolism were downregulated in the control group cows on calving and rapidly upregulated during lactation. The primary bile acid metabolism was upregulated in the exercise group cows on calving compared with that in the control group. Notably, prepartum exercise upregulated tryptophan metabolism during early lactation and downregulated glucose metabolism and the krebs cycle during calving.

### 2.4. Key Metabolites and Correlation Analysis

**Figure 5:**
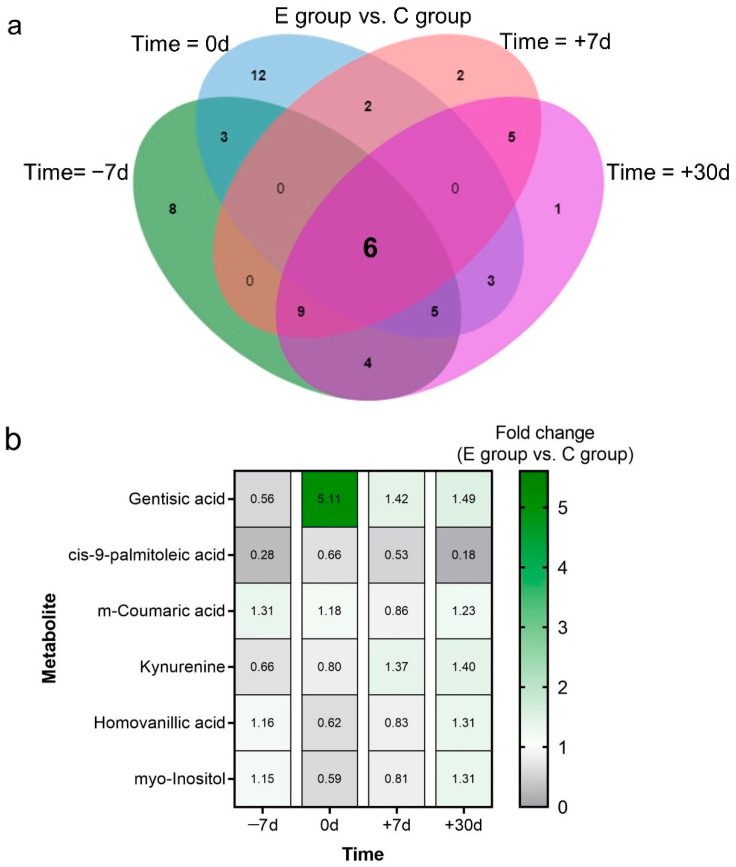
Characterization of key metabolites. (**a**) Venn diagram showing the differential metabolites between the exercise and control groups at −7 d, 0 d, +7 d, and +30 d relative to calving, respectively. (**b**) Fold change in the levels of six shared metabolites between the exercise and control groups at −7 d, 0 d, +7 d, and +30 d relative to calving. A fold change greater than one indicates a relatively higher concentration in the exercise group, whereas a fold change of less than one indicates a lower concentration in the exercise group than in the control group.

The following six metabolites were identified to be commonly changed in the exercise and control groups over the four time points (Figure 5a): gentisic acid, m-coumaric acid, kynurenine, myo-inositol, homovanillic acid, and cis-9-palmitoleic acid (Figure 5b). The cis-9-palmitoleic acid level in cows of the exercise group was consistently lower than that in the control cows. The kynurenine level in cows of the exercise group was low before calving and was high after calving when compared with those in the control cows. The gentisic acid level in cows of the exercise group was higher than that in the control cows after calving. Cross-sectional and longitudinal analysis of differential metabolites revealed three critical metabolites between the exercise and control groups during the transition period including kynurenine, cis-9-palmitoleic acid, and homovanillic acid. The KEGG pathway enrichment analysis revealed the metabolites acting upstream of the pathways of kynurenine, cis-9-palmitoleic acid, and homovanillic acid, including tryptophan, palmitic acid, and dopamine, respectively (Figure 4c).

**Figure 6:**
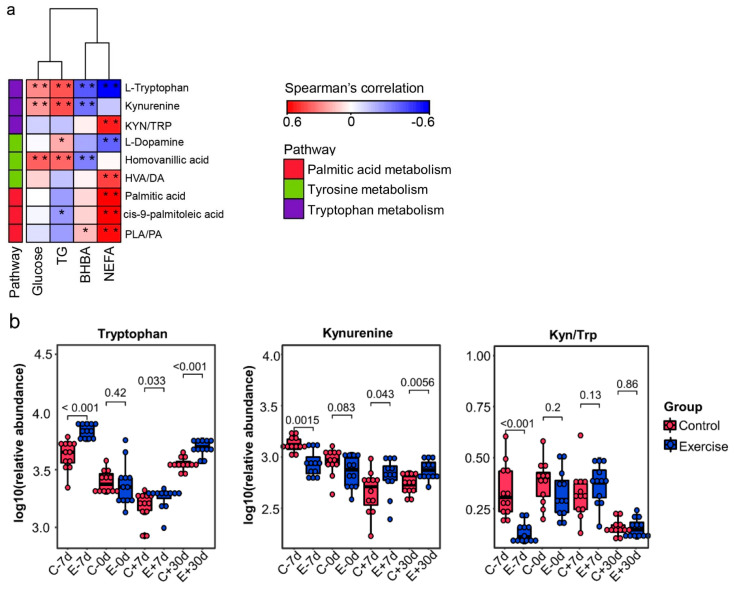
Spearman correlation heatmaps (**a**) for the correlations between phenotypic variables (NEFA, BHBA, TG, and glucose) and key differential metabolites in the exercise and control cows. The scale (right legend) indicates the level of positive (red) or negative (blue) correlations, and asterisks indicate significance: * *p* < 0.05, ** *p* < 0.01. PLA/PA = cis-9-palmitoleic acid to palmitic acid ratio; HVA/DA = homovanillic acid to dopamine ratio; Kyn/TRP = kynurenine to tryptophan ratio; NEFA = non-esterified fatty acids; BHBA = β-hydroxybutyric acid; TG = triglyceride. Box plots (**b**) for the relative abundance of tryptophan and kynurenine, and Kyn/Trp in the exercise and control cows at −7 d, 0 d, +7 d, and +30 d relative to calving.

Spearman correlation analysis showed that the tryptophan, kynurenine, and homovanillic acid levels were negatively correlated (*p* < 0.01) with BHBA, whereas these levels were positively correlated (*p* < 0.01) with glucose and TG (Figure 6a). The tryptophan level was negatively correlated with NEFA, whereas the kynurenine to tryptophan ratio (Kyn/Trp) was positively correlated (*p* < 0.01) with NEFA. The pathway and correlation analyses indicated that tryptophan metabolism may involve lipolysis in cows during the transition period. The tryptophan level in the exercise group cows was high before calving compared with that in the control cows (Figure 6b). However, the kynurenine level and Kyn/Trp ratio in exercise group cows were low before calving compared with those in the control cows (Figure 6b).

**Figure 7:**
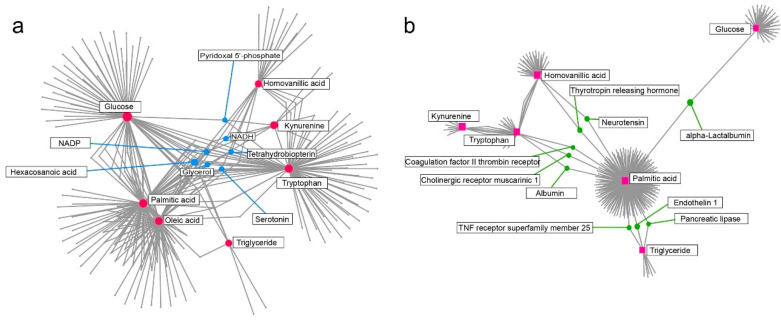
KEGG global metabolic network of key metabolites. (**a**) Metabolite-metabolite interaction network analysis of target metabolites. Known metabolites are presented as red nodes and unknown metabolites are presented as blue nodes. Correlations of metabolites are denoted as edges. NADP = nicotinamide adenine dinucleotide phosphate; NADH = nicotinamide adenine dinucleotide. (**b**) Gene-metabolite interaction network analysis of the target metabolites. Known metabolites are presented as pink nodes and key genes are presented as green nodes. Correlations are denoted as edges.

To further find the potential functional relationships between phenotypic variables and key metabolites, metabolite-metabolite and gene-metabolite interaction network analyses were performed. The interaction network analysis indicated that seven metabolites were associated with glucose, TG, palmitic acid, kynurenine, tryptophan, and homovanillic acid, with hexacosanoic acid, glycerol, and nicotinamide adenine dinucleotide phosphate (NADP) being key components (Figure 7a). Moreover, nine genes were correlated with the target metabolites, including albumin, α-lactalbumin, neurotensin, and pancreatic lipase (Figure 7b).

## 3. Discussion

Dairy cows are challenged by metabolic stress during the transition from late pregnancy to early lactation as characterized by an increase in lipolysis, glucose homeostasis disorder, and inflammatory dysfunction [20,21]. Increased lipid mobilization starts several weeks before calving and is then intensified during calving [22]. In this study, we found that the NEFA plasma level increased from −21 d to calving and then decreased, consistent with the findings of a previous study [23]. Incomplete oxidation of lipids due to their high accumulation produces a large amount of BHBA [5]. NEFA and BHBA are often used as indicators of the risk of peripartum diseases, as they cause a physiological imbalance in transition cows [24]. Here, the prepartum exercise group cows showed decreased concentrations of NEFA in the plasma at −7 d and decreased BHBA concentrations at +7 d relative to calving compared with the control group cows, consistent with the findings of a previous study [15]. The mobilization of NEFA can meet the energy demands of the skeletal muscle, and exercise can increase lipoprotein lipase activity which promotes NEFA uptake by the muscle [25]. Thus, increased utilization of NEFA by the muscle may be responsible for the decreased concentration of NEFA in the plasma with exercise, leading to a decrease in ketone body production. Previous studies have suggested that increased lipolysis may lead to insulin resistance and thus affect glucose homeostasis [26]. We also found that prepartum exercise decreased the concentrations of glucose in plasma on calving compared with those in cows that did not exercise. A previous study showed that high levels of NEFA induced insulin resistance by suppressing insulin signaling in hepatocytes [27]. Archer et al. [28] suggested that exercise may contribute to glucose metabolic homeostasis by inducing the expression of heat shock protein-72 to reduce insulin resistance. Based on these results, prepartum exercise may be associated with reduced insulin resistance to maintain gluconeogenesis on calving; however, further research is needed in this regard. In addition, we found that the TG levels were considerably reduced when the dairy cows entered lactation. The TG level is typically lower during parturition than during pre-calving as prolactin enhances the mammary production of lipids and TGs are a major component of milk [29,30].

Inflammation typically occurs during calving in dairy cows and is also accompanied by increased lipid mobilization [31]. Enhanced lipolysis during the transition period not only increases plasma NEFA levels but also alters fatty acid profiles [6,32]. Palmitic acid and oleic acid are the most abundant saturated fatty acids among NEFA, and high levels of palmitic acid can induce inflammatory responses by activating NF-κB signaling pathways [33]. The present study showed that prepartum exercise considerably decreased palmitic acid and oleic acid levels in cows at −7 d relative to calving, which was consistent with the changes in the NEFA levels. In addition, we found that the palmitoleic acid level and PLA/PA ratio were positively correlated with the NEFA levels. Palmitic acid can be converted to palmitoleic acid via catalysis by stearoyl-coenzyme A desaturase and caffeine can increase the PLA/PA ratio [34]. This was consistent with the changes in the caffeine level and the PLA/PA ratio observed in the exercise group cows. A previous study suggested that palmitoleic acid ameliorated palmitic acid-induced proinflammation via the tumor necrosis factor alpha-independent signaling pathway [35]. Our study also showed that homovanillic acid levels considerably changed in the exercise group cows during the transition period. Previous studies showed that the inflammatory response promoted the conversion of dopamine to homovanillic acid [36,37]. Consistently, we found that the PLA/PA and HVA/DA ratios were positively correlated with the NEFA levels on calving. Therefore, we speculate that exercise might have weakened the inflammatory response during the transition period, although this hypothesis will require further investigation. In addition, we found that primary bile acid biosynthesis decreased considerably during calving and then increased during the lactation period. However, the levels of primary bile acids considerably increased in cows in the exercise group during calving compared with those in the control group. Bile acids are involved in the digestion of food, and dynamic changes in bile acids in the plasma of dairy cows during the transition period also reflect the changes in the DMI [38].

Metabolic changes in transition cows have been described previously [22,39]. Interestingly, we found that tryptophan and kynurenine were critical metabolites in the exercise group cows during the transition period. The tryptophan-kynurenine metabolic pathway has been reported to play an important role in regulating lipolysis, inflammation, and gluconeogenesis [40,41]. NEFA can be displaced for albumin to bind to tryptophan, resulting in lower levels of free tryptophan in the blood [42]. Moreover, phosphodiesterase inhibitors can increase the concentration of NEFA, thereby increasing free tryptophan levels [43]. Consistently, we found that the free tryptophan level negatively correlated with the NEFA levels in the plasma, which increased in cows in the exercise group. The network analysis further indicated that albumin was supposed to be a key link between tryptophan and palmitic acid. Indoleamine 2,3-dioxygenase (IDO) and tryptophan 2,3-dioxygenase are rate-limiting enzymes in the production of kynurenine in the tryptophan degradation pathway [44]. Under an inflammatory status, pro-inflammatory cytokines promote the conversion of tryptophan to kynurenine by upregulating IDO expression [45]. Kynurenine can be converted to 3-hydroxykynurenine by kynurenine monooxygenase which further causes oxidative damage [45]. Our study showed that the Kyn/Trp ratio and NEFA levels were positively correlated during calving, and the Kyn/Trp ratio decreased in cows in the exercise group. These findings suggested that further attention should be paid to elucidating the role of the tryptophan-kynurenine pathway in transition dairy cows. 

Tryptophan is involved in other important pathways where it can directly transform into several molecules by gut microbiota, including indole-3-acetic acid, indole-3-propionic acid, and indole-3-acetaldehyde, which are ligands for aryl hydrocarbon receptor (AhR) and pregnane X receptor [44,46]. The AhR signaling pathway plays a key role in maintaining intestinal barrier integrity and homeostasis [47]. Furthermore, indole can regulate glucose homeostasis by inducing enteroendocrine L-cells to release the glucagon-like peptide-1 [48]. The present study showed that tryptophan and glucose levels were positively correlated and increased in the exercise group cows. Goodarzi et al. [41] suggested that diets supplemented with tryptophan reduced hepatic lipogenesis and gluconeogenesis, and increased glycolysis. Moreover, tryptophan can generate niacin via the kynurenine metabolism pathway; niacin acts to relieve metabolic stress and insulin resistance in periparturient dairy cows [49]. A previous study showed that niacin-supplemented diets reduced TNF-α, haptoglobin, and NEFA concentrations in blood, thus suggesting that niacin inhibited the inflammatory response in dairy cows during early lactation [50]. Therefore, our results suggested that exercise may change the tryptophan metabolism pathway to reduce stress in transition dairy cows, but this remains to be verified.

## 4. Materials and Methods

### 4.1. Animals, Housing, and Management

Animals were treated and samples were collected in strict accordance with the Guidelines for the Care and Use of Laboratory Animals of China, and all procedures were approved by the Animal Care and Use Committee of the Sichuan Agricultural University. The experiment was performed on a dairy farm in Inner Mongolia, China. We selected 24 Holstein transition multiparous dairy cows which were randomly divided into an exercise group (*n* = 12) and a control group (*n* = 12). The cows’ body condition scores, parity, age, and actual gestation days are shown in Supplementary Table S4. The cows were subjected to dry treatment using a cloxacillin suspension for intramammary infusion (Orbenin EDC, Zoetis, NE, USA) at 60 d before the due date, and then transferred to the far-off group (dry-off to 3 weeks before parturition) and close-up group (approximately 21 d before parturition) according to the gestation days. The cows were transferred to maternity stalls when they showed signs of calving and were then transferred to a fresh group after calving. The parturient signs of cows were monitored as described by Proudfoot [51]. The cows were housed in a naturally ventilated barn with 12 rows of head-to-head free stalls and had free access to fresh water. The two doors of the barn provided access to an open-air sports field of 50 m × 45 m. The cows were milked at 06:00, 12:30, and 18:30 and were fed a total mixed ration (TMR) three times a day. The ingredients and chemical compositions of the TMR are listed in Supplementary Table S5.

### 4.2. Experimental Treatments and Sample Collection

The cows in the exercise and control groups were transferred to adjacent close-up pens that had the same facilities and equipment on day −21 of the due date. The structure of the barn and sports field are shown in Supplementary Figure S2. During the experimental period, the sports field was closed off from the control group for an entire day and the cows were allowed to walk around only within the borders of the fence, whereas the cows in the exercise group exercised on the sports field twice daily at 09:00 and 16:00. The entrance to the sports field was closed at all other times. The exercise was started from 20 ± 3 d prepartum until calving. We assisted the cows in walking for a targeted 45 min at 3.25 km/h twice a day. The operation process of driving cattle is described in detail by Black and Krawczel [13].

Before morning feeding, blood samples were collected via the caudal vein at −21 d, −7 d, 0 d, +7 d, and +30 d relative to calving from both the control and exercise cows. Blood was collected on the day of calving (0 d) within 6 h after the appearance of milking colostrum. Plasma was obtained using heparin sodium as an anticoagulant, followed by centrifugation at 1500× *g* for 10 min at 25 °C. All samples were stored at −80 °C.

### 4.3. Plasma Analysis

The concentrations of NEFA, BHBA, glucose, and TG in the plasma were determined using commercially available test kits from the Nanjing Jiancheng Bioengineering Institute, China (#A042–2-1, #E030, #F006-1-1, and #A110-1-1, respectively). All testing procedures were performed in strict accordance with the manufacturer’s instructions.

### 4.4. Sample Pretreatment and Metabolomics Profiling

Following pretreatment, plasma metabolic profiling was performed on a UHPLC system (1290 Infinity II; Agilent Technologies, Santa Clara, CA, USA) coupled to a time-of-flight MS (Agilent 6530; Agilent Technologies, USA) platform. The sample pretreatment, UHPLC-TOF/MS analysis conditions, and equipment parameters used for metabolic profiling are described in detail in the Supplementary Material, section Methods. The feature peak extraction, peak alignment, retention time (RT) correction, and data filtering to extract peak areas were performed using the XCMS package based on the R platform.

### 4.5. Data Processing and Statistical Analysis

Statistical analyses and graphic generation were performed using R software (v4.1, https://www.r-project.org, accessed on 31 March 2021), GraphPad Prism (v9.0, GraphPad, San Diego, CA, USA), Biorender (https://www.biorender.com/, accessed on 10 August 2021), and MetaboAnalyst 5.0 (https://www.metaboanalyst.ca, accessed on 10 August 2021). The total ion peak area was normalized, and after log10 conversion and pareto-scaling processing of the metabolome data, multi-dimensional statistical analysis was performed, including PCA and OPLS-DA. Evaluation of the statistical model and the screening criteria for differential metabolites were consistent with those in our previous study [6]. The Tukey-Kramer adjustment was applied to account for multiple comparisons. A two-tailed Student’s *t*-test was used to evaluate the differences between the exercise and control groups at the same time point. Univariate fold-change analysis was used to compare the relative abundance of metabolites between the control and exercise groups. A variable importance in projection (VIP) value of >1 in OPLS-DA and *p* < 0.05 or 0.05 ≤ *p* < 0.1 were used as the criteria for screening differential metabolites. The threshold of significance was set at *p* < 0.05; trends toward significance were declared at 0.05 ≤ *p* < 0.10. Differential metabolites were identified by comparing the accuracy of m/*z* values (Δ < 25 ppm), and tandem MS spectra were interpreted with reference to available literature and online databases, such as METLIN (https://metlin.scripps.edu/), Bovine Metabolome Database (https://bovinedb.ca/), and MassBank (http://www.massbank.jp/). Moreover, the KEGG pathway analysis and metabolite-metabolite or metabolite-gene interaction networks were constructed using MetaboAnalyst. Spearman’s rank correlation coefficients were calculated to examine the association of phenotypic variables and key differential metabolites using R software. Data are expressed as a mean ± standard error of the mean unless otherwise indicated.

## 5. Conclusions

The metabolism of dairy cows was considerably changed during the transition from late gestation to early lactation, this included increased lipid mobilization and glucose metabolism and decreased bile acid and tryptophan metabolism. Prepartum exercise could decrease the plasma NEFA levels before calving, the glucose level on calving, and the BHBA level after calving. Key differential metabolites and their upstream metabolites, including kynurenine, tryptophan, homovanillic acid, dopamine, cis-9-palmitoleic acid, and palmitic acid, were identified between the exercise and control groups based on an untargeted metabolomics approach. Our findings suggested that prepartum exercise may decrease homovanillic acid and cis-9-palmitoleic acid levels and increase tryptophan levels to alleviate metabolic stress during calving, thereby improving postpartum health. A targeted metabolomics study using chemical standards would be expected to further analyze the metabolites qualitatively and quantitatively.

## Data Availability

Data sharing not applicable.

## Supplementary Materials

The following supporting information can be downloaded at: https://www.mdpi.com/article/10.3390/metabo12040309/s1, Figure S1: Orthogonal partial least square discriminant analysis (OPLS-DA) of scores and permutation test plots, Figure S2: Structure of animal free-stall barns and sports field in the dairy farm in Inner Mongolia Autonomous Region (China). Table S1: Parameters of orthogonal partial least squares discriminant analysis, Table S2: Differential metabolites identified in the control group at −7 d, 0 d, +7 d, and +30 d relative to calving, Table S3: Differential metabolites identified between the exercise and control groups at −7 d, 0 d, +7 d, and +30 d relative to calving, Table S4: Parity, age, body condition score, and gestation days (mean ± SD) of the exercise and control cows during the periparturient period, Table S5: Ingredients and chemical composition of the diets for transition dairy cows.
