# Supplementary material for: Untargeted Metabolomics Reveals Metabolic Stress Alleviation by Prepartum Exercise in Transition Dairy Cows
Source: Metabolites. 2022 Mar 31;12(4):309. doi: 10.3390/metabo12040309 (PMC9028530; doi:10.3390/metabo12040309)
Supplement: Supplementary file 1 [file metabolites-12-00309-s001.zip › metabolites-1584170-supplementary.pdf]

metabolites

Supplementary Materials:

Untargeted Metabolomics Reveals Metabolic Stress Alleviation by Prepartum Exercise in Transition Dairy Cows

Zhengzhong Luo [1,†], Yixin Huang [1,2,†], Li Ma [1], Jing Jiang [1], Qiao Luo [1], Zhuo Yang [3], Kang Yong [4], Liuhong Shen [1], Shumin Yu [1], Xueping Yao [1], Jinzhong Tao [3,*] and Suizhong Cao [1,*]

[1] Department of Clinical Veterinary Medicine, College of Veterinary Medicine, Sichuan Agricultural University, Chengdu 611130, China; zhengzhongluo@163.com (Z.L.); hyxemma213@gmail.com (Y.H.); mali2021202103@126.com (L.M.); jiangjing_94@163.com (J.J.); luoqiao2016@163.com (Q.L.); shenlh@sicau.edu.cn (L.S.); yayushumin@sicau.edu.cn (S.Y.); yaoxueping74@126.com (X.Y.)
[2] Institute of Biodiversity, Animal Health & Comparative Medicine, College of Medical, Veterinary & Life Sciences, University of Glasgow, Glasgow G611QH, UK
[3] Agriculture College, Ningxia University, Yinchuan 750021, China; Jennyyangzhuo@126.com
[4] Department of Animal Husbandry & Veterinary Medicine, College of Animal Science and Technology, Chongqing Three Gorges Vocational College, Chongqing 404100, China; yongkangkang@126.com
* Correspondence: tao_jz@nxu.edu.cn (J.T.); suizhongcao@126.com (S.C.)
† These authors contributed equally to this work.

1. Materials and methods **Page 1**

UHPLC-TOF/MS Analysis

2. List of Figures **Page 2-3**

Figure S1: Orthogonal partial least square discriminant analysis (OPLS-DA) of scores and permutation test plots

Figure S2: Structure of animal free-stall barns and sports field in the dairy farm in Inner Mongolia Autonomous Region (China)

3.List of Tables **Page 4-6**

Table S1: Parameters of orthogonal partial least squares discriminant analysis

Table S2: Table S2: Differential metabolites identified in the control group at –7 d, 0 d, +7 d, and +30 d relative to calving

Table S3: Differential metabolites identified between the exercise and control groups at –7 d, 0 d, +7 d, and +30 d relative to calving

Table S4: Parity, age, body condition score, and gestation days (mean ± SD) of the exercise and control cows during the periparturient period

Table S5: Ingredients and chemical composition of the diets for transition dairy cows

1. Materials and methods

1.1. Sample preparation and UHPLC-TOF/MS Analysis

All plasma samples were thawed at 4 °C, and 100 μL to 300 μL of pre-cooled methanol solution were added. Ten μL of DL-O-Chlorophenylalanine (3.0 mg/mL) was added as an internal standard, vortexed for 30 s and centrifuged at 12,000 r/min for 15 min at 4 °C. Two hundred μL of supernatant was used for test. All samples were mixed in equal volumes (30 μL) to prepare quality control (QC) samples, and seven replicates were set. After pretreatment, plasma samples were separated by using an ultra-high-performance liquid chromatography (UHPLC) system (1290 Infinity II, Agilent Technologies, Santa Clara, CA) incorporating a C18 column (2.1 × 100 mm, 1.8 μm; Agilent). The chromatographic separation conditions were as follows: column temperature 40 °C; flow rate 0.35 mL/min; injection volume 4 μL; mobile phase A: water + 0.1% formic acid, mobile phase B; acetonitrile + 0.1% formic acid. The elution gradient was optimized as follows: 0 min,

95%A and 5% B; 6 min, 80%A and 20% B; 9 min, 50%A and 50% B; 13–16 min, 5%A and 95%B, with a flow rate of 0.4 ml min^{-1}. The injection volume was 10 μL. The samples were placed in the 4 °C autosampler during the whole analysis. All samples were injected randomly, except for a QC sample scattered in every eight samples to monitor the stability of the system and evaluate the reliability of the experimental data.

The plasma samples were analyzed using a quadrupole time-of-flight (TOF) 6530 system (Agilent). The electrospray ionization (ESI) method was selected for mass spectrometry (MS), using nitrogen as the atomizing cone gas, V type flight tube detection mode, and the positive ion (ESI$^+$) and negative ion (ESI$^-$) detection modes. ESI$^+$ mode detection conditions were: capillary voltage 4 kV, sampling cone 35 kV, source temperature 100 °C; desolvation temperature 350 °C, cone gas flow 50 L/h, desolvation gas flow 600 L/h, extraction cone 4 V. ESI$^-$ mode detection conditions were: capillary voltage 3.5 kV, sampling cone 50 kV, source temperature 100 °C; desolvation temperature 350 °C, cone gas flow 50 L/h, desolvation gas flow 700 L/h, extraction cone 4 V. During detection, the data acquisition range was 50–1000 *m/z*, the ion scan time was 0.03 s, and the inter scan time was 0.02 s. In order to ensure the accuracy and repeatability of the detection quality, leucine encephalin (100 ng/ml) was used as the lock mass in positive ($[M+H]^+$, *m/z* 556.2771 Da) and negative ($[M-H]^-$, *m/z* 554.2615 Da) in modes for internal mass calibration. The MS was operated in full scan mode. Data dependent tandem MS was performed on the pooled sample to collect fragmentation spectra for metabolite identification.

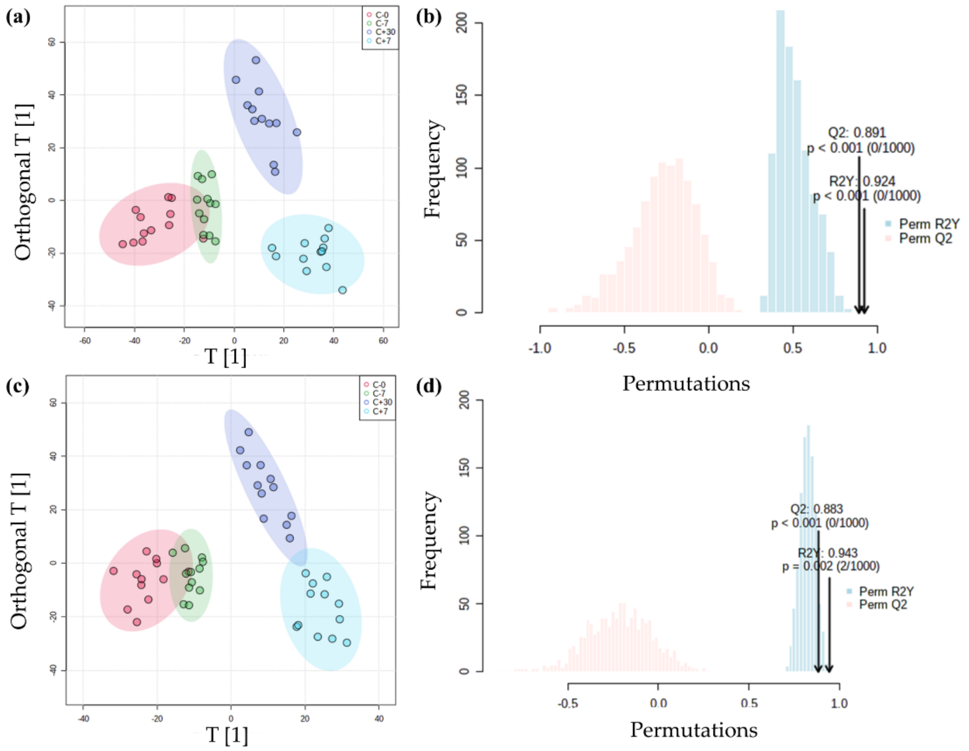

Figure S1. **(a)** and **(b)** Orthogonal partial least square discriminant analysis (OPLS-DA) of scores and permutation test plots for control group on −7 d, 0 d, +7 d, and +30 d relative to calving analyzed in the positive ion mode, respectively. **(c)** and **(d)** OPLS-DA of scores and permutation test plots for control group on −7 d, 0 d, +7 d, and +30 d relative to calving analyzed in the negative ion mode, respectively. T [1] = first principal component. Orthogonal T [1] = second orthogonal component. The 1000-times permutation test of the model showed that the model had high stability.

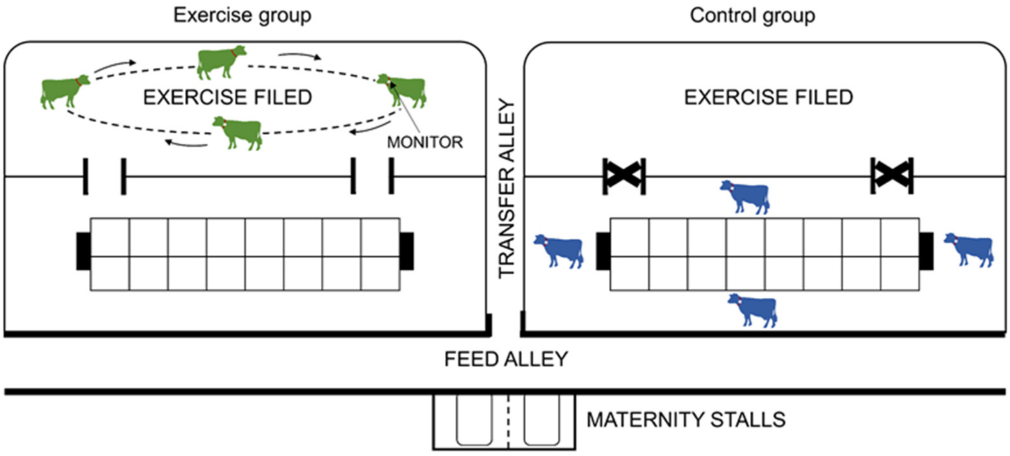

Figure S2. Structure of animal free-stall barns and sports field in the dairy farm in Inner Mongolia Autonomous Region (China). Each cow was equipped with a smart monitor (Allflex, Merck & Co., Inc.,Madison, NJ, USA) on the neck to assess any abnormal behaviors or activities (e.g., fighting, stress, signs of illness).

Table S1. Parameters of orthogonal partial least squares discriminant analysis.

Group	Ion mode	Parameter		
		R^2Y	Q^2	Q^2 intercept[c]
C−7 d vs. T-7 d	ESI+[a]	0.987	0.859	−0.518
C−0 d vs. T-0 d	ESI+	0.987	0.897	−0.506
C+7 d vs. T+7 d	ESI+	0.898	0.550	−0.422
C+30 d vs. T+30 d	ESI+	0.983	0.905	−0.673
C−7 d vs. T-7 d	ESI−[b]	0.997	0.842	−0.353
C−0 d vs. T-0 d	ESI−	0.967	0.857	−0.549
C+7 d vs. T+7 d	ESI−	0.976	0.763	−0.739
C+30 d vs. T+30 d	ESI−	0.992	0.902	−0.571

[a]ESI+, positive ion mode. [b]ESI−, negative ion mode. [c]Q^2 intercept, permutation tests evaluate parameters.ESI+a

Table S2. Differential metabolites identified in the control group at −7 d, 0 d, +7 d and +30 d relative to calving.

No	Metabolite	*m/z*	C-7 d	C-0 d	C+7 d	C+30 d	SEM	-log(*p*)
1	Glycine	76.0394	17.64[a]	19.22[a]	38.71[b]	20.51[a]	2.09	2.14
2	Pyruvate	87.0100	250.87[a]	454.58[b]	150.27[c]	135.05[c]	22.14	5.59
3	β-Alanine	88.0407	16.28[a]	32.42[b]	66.82[c]	54.41[c]	3.50	5.06
4	D-Ribose	149.0460	92.24[a]	56.48[b]	52.72[b]	49.29[b]	3.98	3.30
5	Gentisic acid	153.0192	193.70[a]	63.11[b]	490.62[c]	371.94[c]	31.29	5.25
6	Malic acid	157.0088	108.33[a]	140.15[a]	70.08[b]	52.27[b]	5.55	6.30
7	Gluconic acid	195.0482	245.14[a]	400.53[b]	189.10[c]	182.30[c]	14.44	5.49
8	Acetylcarnitine	204.1188	34.55[a]	27.97[a]	17.12[b]	8.21[c]	2.05	5.64
9	Homovanillic acid	205.0454	160.61[a]	275.68[b]	142.25[a]	79.89[c]	11.68	6.30
10	L-Tryptophan	205.0981	4397.14[a]	2532.87[b]	3651.19[a]	3567.47[a]	149.53	3.54
11	Kynurenine	209.0923	1351.55[a]	938.45[b]	499.51[c]	543.57[c]	57.26	6.05
12	Citric acid	215.0166	674.81[ab]	865.03[a]	270.70[c]	385.87[bc]	47.98	4.71
13	DL-α-Lipoic acid	241.0075	124.98[a]	52.57[b]	76.22[b]	59.29[b]	5.91	3.58
14	Cytidine	244.0927	48.05[a]	55.53[a]	97.85[b]	88.95[b]	6.16	2.26
15	cis-9-palmitoleic acid	255.2326	52.09[a]	172.27[b]	69.94[a]	15.37[c]	10.58	6.19
16	Linoleic acid	279.2311	380.49[a]	1292.19[b]	815.58[c]	441.84[a]	69.16	5.30
17	Arachidonic acid	305.2512	52.35[a]	71.40[b]	41.94[a]	18.45[c]	3.09	6.33

18	Cortisol	397.1699	435.02^{a}	324.19^{b}	197.98^{c}	203.88^{c}	18.09	5.01
19	Maltose	365.1023	246.57^{ab}	1230.68^{c}	365.32b	185.51^{a}	72.87	5.30
20	PGE_1	389.2161	68.68^{a}	24.62^{b}	105.25^{a}	57.35^{a}	6.37	3.51
21	Cholic acid	431.2770	255.97^{ab}	223.97^{bc}	260.71^{ab}	396.63^{a}	20.15	1.94
22	Glycochenodeoxycholic acid	450.3213	311.29^{a}	152.88^{b}	439.12^{a}	662.75^{a}	45.37	3.18
23	LysoPE(0:0/16:0)	454.2945	113.11^{ab}	76.57^{c}	109.20^{b}	146.10^{a}	5.49	3.76
24	LysoPE(0:0/18:3(6Z,9Z,12Z))	474.2611	120.32^{a}	54.07^{b}	99.93^{a}	117.09^{a}	5.92	4.09
25	LysoPC(P-16:0)	480.3426	1433.93^{a}	1570.72^{a}	1562.32^{a}	2905.89^{b}	104.38	4.65
26	Glycocholic acid	488.2984	510.42^{a}	194.38^{b}	562.04^{a}	545.54^{a}	37.85	2.52
27	LysoPC(16:1(9Z))	494.3246	2121.44^{a}	1438.46^{b}	1912.48^{a}	3370.53^{c}	124.39	5.56
28	Tauroursodeoxycholic acid	498.2885	3527.29^{a}	1026.90^{b}	4224.17^{a}	2986.44^{a}	340.09	3.26
29	Taurocholic acid	538.2807	128.79^{a}	55.85^{b}	241.93^{a}	131.41^{a}	16.87	2.91

Note: *m/z*, mass-to-charge ratio. Different letters within a same row indicate significant difference ($p < 0.05$); the same letter indicates no significant difference ($p > 0.05$).

Table S3. Differential metabolites identified between exercise and control groups at −7 d, 0 d, +7 d and +30 d relative to calving.

No.	Metabolite	Fold change[a]			
		T−7 d vs. C-7d	T−0 d vs. C-0 d	T+7 d vs. C+7 d	T+30 d vs. C+30 d
1	Maltose	0.19			
2	cis-9-palmitoleic acid	0.28	0.66^{b}	0.53^{b}	0.18
3	Oleic acid	0.31			0.22
4	Glycocholic acid	0.35	2.81		0.55
5	Cholic acid	0.49			0.62
6	Glycochenodeoxycholic acid	0.49	2.22		
7	Palmitic acid	0.53			0.49^{b}
8	Gentisic acid	0.56	5.11	1.42	1.49
9	LysoPC(14:0)	0.59			
10	Kynurenine	0.66	0.80^{b}	1.37^{b}	1.40
11	Cortisol	0.71			
12	Linoleic acid	0.72		0.74	0.64
13	LysoPC(16:1(9Z))	0.74		1.25	0.73
14	LysoPE(0:0/20:1(11Z))	0.78		1.35	0.77
15	Citric acid	0.79	0.43		
16	Hippuric acid	1.09		0.83	1.22
17	β-Hydroxyisovaleric acid	1.11		0.82	1.24
18	myo-Inositol	1.15	0.59^{b}	0.81	1.31
19	Nonanedioic acid	1.16	1.65		1.15
20	Alpha-ketoisovaleric acid	1.16			
21	Homovanillic acid	1.16	0.62	0.83	1.31
22	Ketoleucine	1.18		0.78	1.21
23	L-Phenylalanine	1.20			1.28
24	m-Coumaric acid	1.31	1.18	0.86	1.23
25	L-Tyrosine	1.33	1.16^{b}		1.26
26	1-Phenylethylamine	1.45		0.80^{b}	1.46
27	Caffeine	1.47			

28	L-Tryptophan	1.58		1.18[b]	1.40
29	Homogentisic acid	1.59	2.07		2.60
30	Xanthine	1.72		1.34[b]	1.26[b]
31	L-Dopamine	1.80	2.07		3.72
32	Glycine	1.81			
33	Trigonellinamide	2.42			
34	PGE_2	5.57	3.13		
35	PGA_2	6.91			
36	Acetylcarnitine		2.15		
37	Allantoin		0.59		
38	Alpha-CEHC			0.78	1.23
39	Capric acid			0.79	1.50
40	Citraconic acid		0.64		1.43
41	Cytidine			2.20	0.46
42	Cytosine		0.79		
43	Dihydrouracil		0.67		
44	D-Ribose 5-phosphate		1.34		1.67
45	Indolelactic acid			0.85	
46	L-Homocitrulline		1.60	-	
47	L-Valine		0.83	0.79	
48	LysoPC(15:0)		1.24	1.31	
49	LysoPC(P-16:0)		-	1.41	0.82
50	LysoPE(0:0/16:0)		1.55		
51	Malic acid		0.70		
52	Pyruvate		0.47		
53	Sebacic acid		1.45		
54	Succinic acid		-	0.81	1.23
55	Taurocholic acid		6.15		
56	Tauroursodeoxycholic acid		4.16		
57	Ubiquinone Q_2		0.25		
58	Uric acid		1.41		1.91
59	Uridine				1.28
60	Vitamin D_3			0.82	

[a]Fold change greater than 1 indicates relatively higher concentration in the exercise group, whereas a fold change of less than 1 indicates a concentration lower than that in the exercise group. [b]The label (b) on the upper right of the number indicates the metabolite that trend towards significantly ($0.05< p <0.1$) differed between exercise and control groups. Unmarked number indicate the metabolite that significantly ($p < 0.05$) differed between exercise and control groups.

Table S4. Parity, age, body condition score, and gestation days (mean ± SD) of exercise and control cows during the periparturient period.

Items	Exercise group (n = 12)	Control group (n = 12)	p-value
Parity	2.25 ± 0.43	2.17± 0.37	0.63
Age (months)	45.71 ± 4.08	43.93 ± 5.44	0.39
Body condition score (prepartum period)	3.56 ± 0.15	3.52 ± 0.22	0.60
Actual gestation days (d)	279.33 ± 3.12	279.00 ± 3.32	0.81

Table S5. Ingredients and chemical composition of the diets for transition dairy cows.

Items	Prepartum	Postpartum

Ingredient, % of DM		
Whole corn silage	15.93	7.79
Oat hay	42.41	
Chinese wildrye	6.2	
Alfalfa hay		18.46
Corn grain	5.99	28.39
Cottonseeds		6.43
Wheat bran	9.09	
Rapeseed meal	3.06	0.83
Soybean meal	9.29	16.78
Cottonseed meal	6.13	2.07
Extruded soybean		6.36
Beet pulp		4.15
Molasses		1.08
Premix[a]	0.37	
Premix[b]		1.24
Magnesium oxide		0.21
Fat powder		2.07
Choline chloride	0.61	-
$Ca_3(PO_4)_2$	0.92	0.62
Calcium chloride		1.04
$NaHCO_3$		1.24
Salt		1.24
Total	100.00	100.00
Chemical composition, % of DM unless noted		
DM	50.9	51.2
CP	15.3	17.3
NDF	47.1	29.8
ADF	28.7	18.0
Starch	9.32	20.3
Ca	0.71	1.04
P	0.37	0.36
NEL, Mcal/kg of DM	1.39	1.82

[a]Formulated to contain (per kilogram of premix) 800,000 IU of vitamin A, 180,000 IU of vitamin D_3, 15,000 IU of vitamin E, 920 mg of vitamin VB_1, 1200 mg of vitamin VB_2, 10 mg of vitamin VB_{12}, 100 mg of D-biotin, 270 mg of D-pantothenic acid, 30,000 mg of nicotinic acid, 1000 mg of Fe, 680 mg of Cu, 1350 mg of Mn, 1800 mg of Zn, 198,400 mg of Mg, 20 mg of Co, and ≤ 10% water. [b]Formulated to contain (per kilogram of premix) 280,000 IU of vitamin A, 100,000 IU of vitamin D_3, 4000 IU of vitamin E, 368 mg of vitamin VB_1, 480 mg of vitamin VB_2, 4 mg of vitamin VB_{12}, 40 mg of D-biotin, 108 mg of D-pantothenic acid, 12,000 mg of nicotinic acid, 744 mg of Fe, 680 mg of Cu, 1612 mg of Mn, 3005 mg of Zn, 31,680 mg of Mg, 24.8 mg of Co, and ≤ 10% water.
